# Pulmonary Resection for Metastasis of Hepatocellular Carcinoma Recurring After Liver Transplant: An Italian Multicenter Experience

**DOI:** 10.3389/fonc.2020.00381

**Published:** 2020-04-15

**Authors:** Federica Invenizzi, Massimo Iavarone, Maria Francesca Donato, Alessandra Mazzucco, Massimo Torre, Serena Conforti, Arianna Rimessi, Claudio Zavaglia, Marco Schiavon, Giovanni Comacchio, Federico Rea, Riccardo Boetto, Umberto Cillo, Daniele Dondossola, Luciano De Carlis, Pietro Lampertico, Mario Nosotti, Paolo Mendogni

**Affiliations:** ^1^Division of Gastroenterology and Hepatology, CRC “A. M. and A. Migliavacca” Center for Liver Disease, Fondazione IRCCS Cà Granda Ospedale Maggiore Policlinico, Università degli Studi di Milano, Milan, Italy; ^2^Thoracic Surgery and Lung Transplant Unit, Fondazione IRCCS Ca' Granda Ospedale Maggiore Policlinico, Milan, Italy; ^3^Thoracic Surgery Unit, Ospedale Niguarda, Milan, Italy; ^4^Hepatology and Gastroenterology Department, Niguarda Ca' Granda Hospital, Milan, Italy; ^5^Department of Cardiac, Thoracic, Vascular Sciences and Public Health, Padua University Hospital, Padua, Italy; ^6^Department of Surgery, Oncology and Gastroenterology, Hepatobiliary Surgery and Liver Transplantation, Padua University Hospital, Padua, Italy; ^7^HBP Surgery and Liver Transplantation Unit, Fondazione IRCCS Ca' Granda Maggiore Hospital, University of Milan, Milan, Italy; ^8^Department of General Surgery and Transplantation, Niguarda Ca' Granda Hospital, Milan, Italy; ^9^University of Milan, Milan, Italy; ^10^Department of Pathophysiology and Transplantation, Università degli Studi di Milano, Milan, Italy

**Keywords:** hepatocellular carcinoma, liver transplantation, recurrence, pulmonary metastases, pulmonary resection

## Abstract

**Background and aim:** Liver transplantation (LT) is a validated treatment for hepatocellular carcinoma (HCC). HCC recurrence occurred between 8 and 20% of patients and lung is the most frequent site. Pulmonary metastases resection (PMR) prolongs survival, however in LT-setting the impact on survival is unclear. To give new lights on this issue, we report the experience of three Italian LT Centers.

**Methods:** All consecutive HCC transplanted patients in three Italian LT Centers, who developed pulmonary metastasis from HCC (PM-HCC), as first metastasis, from 2008 to 2018, were included whenever treated with PMR.

**Results:** Twenty-five patients were enrolled (median age 58 yrs, 84% male, 3% cirrhotics). HCC recurred after 34 months (9–306) since LT and PMR was performed after 2.4 months (0–43.1). A total of 28 PMR (19 single resections; 9 multiple resections; 16 right; 2 left) have been performed on 24 patients while in one case percutaneous microwave ablation (MWA) was preferred. Four patients have been re-operated due to pulmonary HCC-recurrence after surgery. The majority of surgical resection type was wedge resection (26, 89%). Surgical access was: video-assisted thoracic surgery (VATS) in 17 cases (59%); thoracotomy in 11 (38%); MWA in 1 (3%). The 48% of nodule was in right lower lobe. Perioperative in-hospital mortality and 30 days mortality were nil; median surgical time 90 min (50–365); median post-operative overall stay 5 days (2–11). Post-operative ICU treatment was necessary in 1 case (3%) for 3 days; blood transfusions in 2 cases (7%). Overall, 5 complications (2 bleeding; 1 AKI; 1 major cardiac; 1 wound dehiscence) occurred, with an overall complications rate of 23%. Eight (32%) patients died during a follow-up after HCC recurrence of 32 months (7–213): 7 for HCC progression, 1 for severe liver failure due to chronic rejection. The 1 and 5 year cumulative probability of OS from recurrence were 100 and 43% (95%CI 12–74), respectively, with a median OS of 51 months (95%CI 24–78).

**Conclusion:** Selected patients with isolated pulmonary HCC-recurrence after LT and with preserved hepatic function showed that a pulmonary metastasectomy could be efficacious in managing a PM-HCC and could give an opportunity for long-term survival.

## Introduction

Liver transplantation (LT) is a validated treatment for hepatocellular carcinoma (HCC) (1, 2). The risk of HCC recurrence varies between 8 and 20% depending on pre-transplant-variables such as the tumor burden and the alpha-fetoprotein (AFP) level (3–7). Moreover, HCC recurrence after LT is an accepted and not exceptional clinical challenge (8). In fact, it reduces LT curative intention, being the survival reduced as low as 3.3 months when only best supportive care is offered (9). Surveillance and administration of HCC recurrence after LT is exciting and complicated. More frequently, recurrence occurs within the first 2 years (10), although cases of very late recurrence after a 10 year period have been reported (11, 12).

Regarding the sites of HCC recurrence, the first revelation is more frequently extra-hepatic (as high as 71%), followed by intra-hepatic and simultaneously intra- and extra-hepatic (13). In addition, intra- or extrahepatic recurrence, or the combination of both, did not result in different outcomes. Among the extrahepatic sites of recurrence, the lung is the most frequent metastatic site (5). Pulmonary metastases resection (PMR) for HCC improves survival in patients treated by liver resection or ablative procedures (14). Although some investigators recommend PMR also for HCC recurrence after LT (15, 16), in this setting the impact on survival is unclear and today pulmonary metastases for HCC (PM-HCC) after LT are infrequently resected due to their multiplicity and multi-organ involvement.

To give new lights on this issue, we report the experience of three Italian LT Centers regarding pulmonary metastasectomy for HCC after LT in terms of safety, complication, risk of recurrence, and survival.

## Patients and Methods

### Study Design, Patients, and Endpoints

This is a retrospective evaluation of a cohort of prospectively enrolled patients in three different Liver Transplant Centers in Italy (two in Milan and one in Padua). All consecutive HCC transplanted patients, who developed PM-HCC, as first metastasis, from 2008 to 2018 were included in this study whenever treated with lung surgery resection. The database was locked in June 2019. Baseline corresponded to first HCC recurrence after LT with lung metastases. Each patient signed a written informed consent in conformity with the ethic committee and the ethical guidelines of the 1975 Declaration of Helsinki, as updated in 2004.

A contrast-enhanced thoraco-abdominal computed tomography (CT)-scan was performed every 6 months for the first 5 years, and then annually in all HCC transplanted patients. Serum AFP was quantified every 3 months during the first year and every 6 months thereafter. In case of suspected HCC recurrence, biopsy was performed when the site of recurrence is reachable. Otherwise, the diagnosis was obtained by imaging associated with AFP levels increase. Once the diagnosis was obtained, surgery treatment was performed whenever judged appropriate, with a curative or palliative intent. For the analysis, treatments were considered with a curative intent if the removal of the neoplastic lesion led the patient HCC-free.

Once the diagnosis was obtained, in one of the three centers sorafenib was started as soon as became available and its use safe (i.e., after healing of surgical wound in surgical patients) and mammalian target of rapamycin inhibitor (mTORi)-based immunosuppression regimen (sirolimus or everolimus) was considered.

The following data were collected: demographics and pre-LT history, native liver histological tumor staging, immunosuppressive regimens, time, characteristics and treatments of HCC recurrence, surgery procedures, and complications.

The primary endpoint was safety of surgery on lung metastasis in HCC recurrent patients after LT. Secondary endpoints were feasibility of surgery in these patients, recurrence after the first surgical treatment and survival.

Surgery safety was assessed with evaluations of multiple parameters: perioperative in-hospital mortality (considered as death for any causes during post-operative in-hospital course), 30 days mortality (considered as death for any causes within 30 days after thoracic surgery), surgical skin-to-skin time, post-operative hospital stay, need of post-operative intensive care unit (ICU), blood transfusions, readmission rate, post-operative complications. We considered as complication any of the following: bleeding, pneumonia, major cardiac complications (atrial fibrillation or acute myocardial ischemia), persistent air leak >7 days, wound infection or dehiscence, acute kidney injury (AKI). A CT-scan every 2 months after surgery and during follow-up was used to assess tumor response, according to modified Response Evaluation Criteria in Solid Tumors (RECIST) criteria (17). Time to radiological progression was defined as the time elapsed from baseline to disease progression according to modified RECIST criteria for HCC. Overall survival (OS) was measured from the date of first surgical intervention until the date of death from any cause or date of the last visit. Baseline variables were also analyzed in order to identify predictors of OS.

### Surgery

Patients with suspected or diagnosed thoracic metastasis from HCC have been referred to thoracic surgery department. Firstly, all referred patients were screened and judged suitable or unsuitable for metastasectomy from surgical anatomical point of view. Considering the metastatic nature of the thoracic nodules, the intervention proposed was a minimally invasive lung-sparing resection, whenever possible. If positively judged for surgery, patients were functionally evaluated by cardio-pulmonary tests and, finally an anathesiological risk was formulated, according to the American Society of Anesthesiologists (ASA) physical status classification system. The intraoperative and post-operative management was carried out in a standard way.

### Statistical Analysis

We used standard statistics (median and range for continuous variables, percentage for categorical variables) to describe baseline series characteristics and safety data. Survival time was computed as the interval between first HCC recurrence and death (survival after recurrence). Survival time was censored at the date of last contact in living patients. Survival curves were estimated with the non-parametric Kaplan-Meier method. Calculations were done using SPSS Statistics Program.

## Results

Twenty-five patients were enrolled. Patients' baseline characteristics are shown in Table 1. HCC recurred after a median of 33.6 months (8.9–306.4) since LT. PM-HCC treatment was performed after a median of 2.4 months (0–43.1) following HCC recurrence. A total of 28 PMR (19 single resections; 9 multiple resections; 16 right; 2 left) have been performed on the 24 enrolled patients. In additional patient, transthoracic percutaneous microwave thermoablation (MWA) was preferred, due to the high risk for surgery. Four patients have been re-operated due to pulmonary HCC-recurrence after the first PMR. The majority of surgical resection type was wedge resection (*n* = 26, 89.2%), while only one segmentectomy and one lobectomy were performed. The surgical access was as follows: video-assisted thoracic surgery (VATS) in 17 cases (58.6%); thoracotomy (11 cases, 37.9%); MWA (1 case, 3.4%). None conversion from VATS to thoracotomy was needed. The presence of pleural adhesions was observed in 7 cases (24.1%). The average of nodules treated per procedure was 1.5 (1–4). The majority of nodule treated was in right lower lobe (12), followed by left lower lobe (7), left upper lobe (6), right upper lobe (4), and right middle lobe (3). In Table 2 per-patient's characteristics were presented.

**Table 1:** Baseline features of the 25 patients enrolled in the study.

**Features**	**Overall (*n* = 25)**
Age, years^*^	58 (41–73)
Male, N.	21 (84%)
Smoke, N.	8 (32%)
COPD, N.	5 (20%)
FEV1, % of predicted^*^	91 (76.1–124)
CAD, N.	0
Diabetes, N.	2 (8%)
BMI, kg/m^2^^*^	27 (19–30)
Anticoagulant or antiplatelet drugs, N.	6 (24%)
Cirrhosis, N.	3 (12%)
Liver disease etiology, N.	
HCV	13 (52%)
HBV	6 (24%)
Other etiologies	6 (24%)
Native liver histology, N.	
Milan-in	12 (48%)
Microvascular invasion	11 (44%)
Edmonson grade 3 or 4	9 (36%)
HCC recurrence time, months^*^	34 (9–306)
HCC recurrence pattern, N.	
Liver only	0
Intra and extra-hepatic	0
Extra-hepatic only	25 (100%)
AFP levels, ng/mL^*^	7 (1–85)
Immunosoppressive regimen, N.	
CNi+mTORi	4 (16%)
mTORi	3 (12%)
CNi	11 (44%)
CNi+MMF	7 (28%)

* *Median (range); COPD, chronic obstructive pulmonary disease; FEV1, forced expiratory volume at 1 s; CAD, coronary artery disease; BMI, body mass index, HCV, hepatitis C virus; HBV, hepatitis B virus; HCC, hepatocellular carcinoma; AFP, alpha-fetoprotein; CNi, calcineurine inhibitor; mTORi, mammalian target of rapamycin inhibitor; MMF, mycofenolate*.

**Table 2:** Individual surgery details and follow-up of the 25 patients enrolled in the study.

**Patients**	**Gender, age (years)**	**Time to recurrence from OLT (months)**	**Nodules: number/size (mm)**	**PET scan**	**Resection**	**Lobe**	**Surgical access**	**ICU**	**Blood trasfusion**	**Surgery complications, type**	**HCC recurrence after surgery**	**Time of recurrence from surgery (months)**	**Site of recurrence after surgery**	**Polmonary surgery for recurrence**	**Follow-up (months)**	**Dead**
#1	Male, 62	92	1/18	Pos	WEDGE	LUL	VATS	No	No	No	Yes	8.7	Abdomen	No	50	No
#2	Male, 57	57	1/NA	NA	MWA	LUL	VATS	No	No	No	**	NA	NA	NA	39	Yes
#3	Male, 58	13	1/13	Neg	WEDGE	RLL	VATS	No	No	No	Yes	4.1	Lung	No	97	No
#4	Female, 60	34	1/NA	Pos	WEDGE	RUL	VATS	No	No	Yes, wound infection/dehiscence	No	NA	NA	NA	16	No
#5	Male, 61	18	1/12	Neg	WEDGE	LLL	VATS	No	No	No	**	NA	NA	NA	26	Yes
#6	Male, 58	25	1/5	Neg	WEDGE	RLL	VATS	No	No	No	Yes	20.9	Lung	Yes	39	Yes
#7	Male, 41	9	3/NA	NA	WEDGE	RLL, RUL	Thoracotomy	No	Yes	Yes, haemothorax	Yes	9.2	Multifocal	No	31	Yes
#8	Male, 51	13	1/17	Neg	WEDGE	LUL	VATS	No	No	No	Yes	16.3	Liver	No	29	Yes
#9	Male, 53	15	1/4	Pos	WEDGE	RUL	Thoracotomy	No	No	No	No	NA	NA	NA	23	No
#10	Male, 60	9	2/8	NA	WEDGE	LLL	VATS	No	No	No	Yes	5.3	Bone	No	7	No
#11	Female, 43	36	3/7	Pos	WEDGE	LLL	Thoracotomy	No	No	No	Yes	2.1	Lung	Yes	213	No
#12	Female, 71	306	2/4	Pos	WEDGE	RLL	Thoracotomy	Yes,3 days	No	Yes, acute renal failure	No	NA	NA	NA	19	No
#13	Male, 62	62	1/12	Neg	SEGMENTECTOMY	RLL	VATS	No	Yes	Yes, haemothorax	No	NA	NA	NA	8	No
#14	Male, 68	42	1/17	Neg	WEDGE	LLL	VATS	No	No	No	Yes	1.5	Multifocal	No	47	No
#15	Male, 64	1	1/10	NA	WEDGE	LUL	VATS	No	No	No	Yes	23.7	Multifocal	No	33	Yes
#16	Male, 68	8	1/13	NA	WEDGE	RLL	VATS	No	No	No	No	NA	NA	NA	32	No
#17	Male, 49	6	2/12	NA	WEDGE	RLL	VATS	No	No	No	No	NA	NA	NA	32	No
#18	Male, 51	51	1/33	Pos	WEDGE	ML	Thoracotomy	No	No	No	Yes	14.5	Multifocal	No	21	Yes
#19	Male, 68	36	2/15	Pos	WEDGE	LLL, LUL	Thoracotomy	No	No	No	Yes	15	Lung	No	51	Yes
#20	Male, 70	67	1/14	Neg	LOBECTOMY	ML	VATS	No	No	No	Yes	5.3	Liver	No	57	No
#21	Male, 56	50	1/6	Neg	WEDGE	RLL	VATS	No	No	No	No	NA	NA	NA	36	No
#22	Male, 52	14	1/22	Pos	WEDGE	RLL	Thoracotomy	No	No	No	Yes	32.6	Lung	Yes	35	No
#23	Male, 53	20	1/13	Pos	WEDGE	RLL	Thoracotomy	No	No	No	No	NA	NA	NA	12	No
#24	Male, 58	46	3/15	Neg	WEDGE	LLL	VATS	No	No	No	No	NA	NA	NA	18	No
#25	Male, 73	121	2/15	Pos	WEDGE	LLL	Thoracotomy	No	No	No	No	NA	NA	NA	6	No

*LLL, left lower lobe; LUL, left upper lobe; PET, positron emission tomography; LM, median lobe; RLL, right lower lobe; RUL, right upper lobe; ICU, intensive care unit*.

VATS, video-assisted thoracic surgery; MWA, percutaneous microwave ablation;

** *Diagnostic surgery; Pos, positive; Neg, negative; NA, not available or not applicable*.

### Safety Analysis

Perioperative in-hospital mortality and 30 days mortality were nil. Median surgical time (skin-to-skin) was 90 min (range 50–365); median post-operative overall stay was 5 days (range 2–11). Post-operative ICU treatment was necessary in 1 case only (3.4%), for 3 days. Blood transfusions were necessary in 2 cases (6.9%). Readmission was required in 1 case (3.4%). A total of 5 complications (2 bleeding; 1 AKI; 1 major cardiac; 1 wound dehiscence) were observed, with an overall complications occurrence of 22.7% (detailed in Table 2).

### Effectiveness and Survival

**Figure 1:**
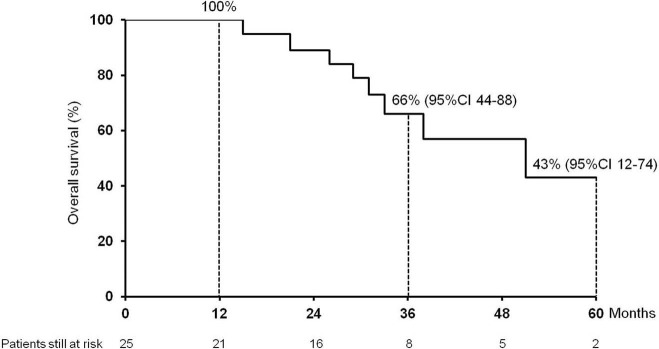
Cumulative survival (1, 3, and 5 year) of the 25 patients enrolled in the study after hepatocellular carcinoma recurrence following liver transplantation.

The median follow-up after HCC recurrence was 32.5 months (7.4–213.0). Eight (32%) patients died: 7 for HCC progression, 1 patient for severe liver failure due to chronic rejection. The 1-, 3-, and 5 year cumulative probability of OS from recurrence were 100, 66% (95%IC 44–88) and 43% (95%CI 12–74), respectively, with a median OS of 51 months (95%CI 24–78) (Figure 1).

## Discussion

This is the first study, to the best of our knowledge, to specifically evaluate the impact and management of lung metastases in HCC recurrence after LT. We observed a low occurrence of complications, no surgery-related mortality and a good feasibility of surgery in those patients judged operable after first RPM whenever HCC recurred in lung. We observed that the majority of recurrences were in the right lower lobe. This is probably due to the proximity of this site to the liver at the time of hepatectomy and to the lymphatic drainage. Finally, we detected good long-term survival outcomes, with a 1-, 3-, and 5 year cumulative probability of OS from recurrence of 100%, 66% (95%CI 44–88) and 43% (95%CI 12–74), respectively, and a median OS of 51 months (95%CI 24–78).

Our study would have a great impact in the setting of LT for HCC, since the second most common site of HCC recurrence is the lung. Whenever the lesions were limited to the lung, we were able to show that complete resection can be safely performed, which may have contributed to the long-term survival of these patients, while some reviews advocated more aggressive surgical intervention (5, 6). This is of great importance, since tumor recurrence would have obviously decreased the opportunity of long-term survival after LT for HCC (6, 15). To the best of our knowledge, only few reports of resection for pulmonary metastasis from HCC after liver transplantation are available (15, 16, 18–22). In particular, Bates et al. (16) reported five cases of metastatic HCC resection after liver transplantation, with a survival period of 44 months after LT and 28 months after pulmonary resection.

Finally, our results in terms of OS are comparable to those of patients following metastasectomy after liver resection for HCC in patients without history of LT (14, 23–25).

Although prognostic indicators are not known, several clinical or pathological factors have been published (14, 23, 26, 27). Generally, patient selection and metastasis characteristics (such as number and side of lesions) are indispensable for a surgery survival benefit. In additional, good liver function might be essential for better survival (28, 29). As confirmation of this, in our study population was well-selected (only lung metastasis) with a good liver function (no patients with decompensated disease).

After the metastasectomy, recurrence may be managed by a repeated metastasectomy (23, 30–32). In our series, 4 patients (3 cases for recurrence, once case to complete the resection) underwent to a second pulmonary resection for a recurrent PM-HCC after a metastasectomy. Multiple metastases or repeated metastasectomies are not risk factors for long-term survival, as shown in this and previous studies (31, 33), although patients with a single metastasis or single pulmonary surgery might show better survival (23, 26).

Limitations of our study are: the retrospective design, the lack of a control arm and the limited number of patients. The impact of surgical access on outcome cannot be assessed, due to the limited number of patients. However, the study has several strengths: this is the largest consecutive series of patients affected by lung HCC-metastasis treated surgery in the post-transplant setting. Moreover, despite the retrospective design of the study, the cohort is homogeneous regarding the baseline features and the patient management.

In conclusion, selected patients without intrahepatic HCC-recurrence after LT and with preserved hepatic function showed that a pulmonary metastasectomy could be efficacious in managing a PM-HCC and could give an opportunity for long-term survival.

## Data Availability Statement

The datasets generated for this study are available on request to the corresponding author.

## Ethics Statement

Ethical approval was not provided for this study on human participants because this is a retrospective. The patients/participants provided their written informed consent to participate in this study.

## Author Contributions

FI, MI, and PM planned the study, analyzed and interpreted the data, and wrote the manuscript. MN and UC participated in the analyzing of data and writing of the manuscript. CZ, MD, MN, PL, and LD critically revised the manuscript for important intellectual content. FI, MI, AM, MT, SC, AR, CZ, MS, GC, FR, RB, DD, and PM enrolled patients and acquired data.

### Conflict of Interest

FI is a member of a speakers bureau for Abbvie and Gilead Science. MI received speaking and teaching fees from Bayer, Gilead Science, Janssen, BTG, and Abbvie, and is a consultant for BTG. PL is a member of a speakers bureau for BMS, Roche, Gilead Sciences, GSK, MSD, and Abbvie, and is on an advisory board for Janssen, Eiger, and Myr pharma. MFD is a member of a speakers bureau for BMS, Abbvie, and MSD. The remaining authors declare that the research was conducted in the absence of any commercial or financial relationships that could be construed as a potential conflict of interest.
